# Saturation Mutagenesis for Phenylalanine Ammonia Lyases of Enhanced Catalytic Properties

**DOI:** 10.3390/biom10060838

**Published:** 2020-05-30

**Authors:** Raluca Bianca Tomoiagă, Souad Diana Tork, Ilka Horváth, Alina Filip, Levente Csaba Nagy, László Csaba Bencze

**Affiliations:** Biocatalysis and Biotransformation Research Center, Faculty of Chemistry and Chemical Engineering, Babeș-Bolyai University, Arany János street 11, RO-400028 Cluj-Napoca, Romania; tomoralu15@gmail.com (R.B.T.); ida.souad@gmail.com (S.D.T.); horvath_ilka@yahoo.com (I.H.); afilip@chem.ubbcluj.ro (A.F.); nc35@chem.ubbcluj.ro (L.C.N.)

**Keywords:** biocatalysis, phenylalanine ammonia-lyases, saturation mutagenesis, protein engineering

## Abstract

Phenylalanine ammonia-lyases (PALs) are attractive biocatalysts for the stereoselective synthesis of non-natural phenylalanines. The rational design of PALs with extended substrate scope, highlighted the substrate specificity-modulator role of residue I460 of *Petroselinum crispum* PAL. Herein, saturation mutagenesis at key residue I460 was performed in order to identify *Pc*PAL variants of enhanced activity or to validate the superior catalytic properties of the rationally explored I460V *Pc*PAL compared with the other possible mutant variants. After optimizations, the saturation mutagenesis employing the NNK-degeneracy generated a high-quality transformant library. For high-throughput enzyme-activity screens of the mutant library, a PAL-activity assay was developed, allowing the identification of hits showing activity in the reaction of non-natural substrate, *p*-MeO-phenylalanine. Among the hits, besides the known I460V *Pc*PAL, several mutants were identified, and their increased catalytic efficiency was confirmed by biotransformations using whole-cells or purified PAL-biocatalysts. Variants I460T and I460S were superior to I460V-*Pc*PAL in terms of catalytic efficiency within the reaction of *p*-MeO-Phe. Moreover, I460T *Pc*PAL maintained the high specificity constant of the *wild-type* enzyme for the natural substrate, l-Phe. Molecular docking supported the favorable substrate orientation of *p*-MeO-cinnamic acid within the active site of I460T variant, similarly as shown earlier for I460V *Pc*PAL (PDB ID: 6RGS).

## 1. Introduction

Phenylalanine ammonia-lyases (PALs, EC 4.3.1.24, and PAL/TALs with combined phenylalanine and tyrosine ammonia-lyase activities, EC 4.3.1.25) play essential roles in the non-oxidative ammonia elimination of phenylalanine to cinnamic acid, an important precursor for the biosynthesis of phenylpropanoids. From a synthetic point of view, PALs, under high ammonia concentration, can perform the reverse ammonia addition reaction onto α,β-unsaturated arylacrylates, to produce l-α-amino acids in high enantiomeric purity. Accordingly, PALs from *Rhodotorula glutinis* (*Rg*PAL) [1,2], *Petroselinum crispum* (*Pc*PAL) [3], and *Anabaena variabilis* (*Av*PAL) [4] due to their broad substrate tolerance emerged as efficient biocatalysts for the production of phenylalanine analogs [5,6,7,8,9,10]. Recent protein engineering increased the catalytic activity [11,12,13,14] and/or extended the substrate scope of PALs [15,16,17]. Despite these advances, the broader application of PALs for the large-scale synthesis of various specific target molecules still remains a challenge due to the substrate/product inhibition [14,18] and/or enzyme stability [16,19,20] related issues, especially in the synthetically attractive ammonia addition reaction, that requires high (4–6 M) ammonia concentrations.

Directed evolution driven protein engineering is one of the most powerful tools to extend the substrate scope of enzymes, or to increase their catalytic activity, but also to alleviate the substrate/product inhibition or protein stability related dilemma [21,22,23,24]. The bottleneck of the directed evolution is the screening effort, necessary for the proper selection of hits from the largely sized mutant libraries (> 10^3^–10^4^ clones), that requires high-throughput enzyme activity assays applicable at whole cell/cell lysate level. Among the PAL-activity assays, the high-performance liquid chromatography (HPLC) based procedure [14,16] allows the determination of conversion and enantiomeric excess values but can only be employed for the screening of limited sized libraries derived from rational design mutagenesis or single combinatorial active-site testing (CAST) [25]. The UV-spectroscopy based PAL-activity assay monitors the production or consumption of the cinnamic acid derivatives [26,27] and possesses low sensitivity in the case of using cellular biocatalysts. Moreover, the reported fluorescence PAL-activity assays, despite their sensitivity, are limited for activity tests using the non-natural substrate *o*-NH_2_-phenylalanine [28] or require high instrumentation facilities, such as inductively coupled plasma–mass spectrometry (ICP-MS) [29], thus their general applicability is suppressed. Another important issue is the quality of the transformant library, obtained through different mutagenesis methods, high-quality libraries, with a higher frequency of hits, providing the advantage of a reduced screening effort. In this context, iterative saturation mutagenesis (ISM) [30,31] has emerged as an effective tool, where multiple protein sites composed of one or more amino acid residues are chosen for randomization through saturation mutagenesis, while the obtained hits are used as templates to perform randomization at the other sites in iterative cycles until the desired degree of catalyst improvement is reached. Combinatorial active-site saturation test (CAST) employs the methodology of ISM for residues within the catalytic site, creating smaller, high-quality libraries [25,32,33].

For phenylalanine ammonia-lyases (PALs), limited directed evolution and/or saturation mutagenesis studies have been performed, focusing on inducing d-selectivity [34] or increased activity towards *p*-substituted bulky [12,13] or electron donor substrates [17]. Modification of the catalytic site of *Pc*PAL through smaller amino acid substitutions of the active site residues I460 or/and F137, extended its substrate scope towards bulky phenylalanine analogs [16]. Furthermore, mapping the hydrophobic region of the active site of *Pc*PAL [14] revealed the importance of residue I460 for the enzyme activity modulation towards substrates bearing bulky substituents mostly in *m*- or *p*-positions on the aromatic ring. Based on the above, the synthetic utility of I460V variant was also demonstrated by the production of several *m*-, or *p*-substituted phenylalanine analogs of high synthetic value, employing biotransformations catalyzed efficiently by mutant I460V, but with low efficiency by *wild-type Pc*PAL [18].

In this context, this study focused on the randomization by site-saturation mutagenesis of the key activity modulator residue I460 of *Pc*PAL in order to identify novel mutant variants with increased activity towards selected substrates of synthetic interest. For obtaining high-quality libraries, with increased frequency of hits within the transformant library, the mutagenesis procedure was optimized, while the PAL-activity assay was adapted to allow the high-throughput activity screens of cell lysates providing candidates of which superior activity was validated.

## 2. Materials and Methods

### 2.1. Saturation Mutagenesis Procedure:

Site-saturation mutagenesis experiments were carried out using as template the pET19b vector harboring the gene encoding PAL from Petroselinum crispum [35]. The primers containing the NNK degenerate codons (Tables S1 and S2) were purchased from Invitrogen (Karlsruhe, Germany).

#### 2.1.1. Procedure Using Megaprimers:

PCR reactions were performed in a total volume of 50 μL, using 100 ng template DNA, 0.1 μM NNK degenerate (forward) and flanking (reverse) primer, 0.1 μM dNTPs, 1 U Phusion High Fidelity polymerase. The PCR protocol consisted of initial denaturation for 3 min at 95 °C followed by 10 cycles of 30 s at 95 °C, 1 min at 64 °C annealing temperature, established by initial gradient PCR experiments and extension for 1 min at 72 °C. The second stage PCR consisted of 25 cycles of 30 s at 95 °C and extension for 8 min at 72 °C; followed by a final extension of 16 min at 72 °C.

#### 2.1.2. Procedure Using Partially Overlapping Primers:

The PCR reactions for the overlapping method used 2 ng template, 2 μM of each primer, 0.1 μM dNTPs, 1 U Phusion High Fidelity polymerase in a total volume of 50 μL. The PCR protocol consisted of: initial denaturation for 3 min at 95 °C followed by 30 cycles of 1 min at 95 °C, annealing 1 min at T_m no_-5 °C and extension 15 min at 72 °C, followed by final denaturation at 95 °C for 1 min, annealing at T_m pp_-5 °C for 1 min and extension for 15 min at 72 °C.

The PCR products from both procedures were digested with DpnI (overnight, 37 °C) and purified through ethanol precipitation. 5 μL of the purified PCR product was electroporated into 50 μL *E. coli* BL21 GOLD (DE3) electrocompetent cells, followed by incubation in SOC medium for 1 h, at 37 °C. The mixture was plated on Luria Bertani (LB) agar plates supplemented with carbenicillin (50 μg/mL) and incubated overnight at 37 °C, obtaining the corresponding transformant libraries.

### 2.2. Activity Screens of Transformant Libraries

The colonies of the transformant libraries (113 colonies from each library) were picked and transferred to 100 µL carbenicillin supplemented (50 μg/mL) LB-medium distributed in the wells of a 96-deep-well polypropylene plate, followed by overnight incubation at 37 °C, 200 rpm. 8 μL of each preculture were used to inoculate 800 μL of LB-medium distributed within a 96-deep-well plate, followed by cell growth and induction at OD_600_ 0.5–0.7 using 0.5 mM IPTG. The colonies were grown to an OD_600_ of ~2 (corresponding to a wet cell concentration of ~12 mg/mL) by incubation at 25 °C, 200 rpm for 20–24 h. The cultures were pelleted by centrifugation, 20 min, 4000 rpm (1751× *g*) at 4 °C, and the cell pellets were deposited at −80 °C prior to their use.

For activity screens, each cell pellet was resuspended in 400 μL lysis buffer (20 mM PBS pH = 7.00, NaCl 50 mM, 0.07 mM lysozyme, 0.36 mM PMBS), followed by incubation for 40 min, 800 rpm at 30 °C, obtaining by centrifugation (20 min, 4000 rpm or 1751× *g*, 4 °C) the clarified lysates as supernatant. The cell lysates of the PAL variants of the transformant libraries were assayed using racemic *p*-methoxyphenylalanine as substrate monitoring the production of *p*-methoxy *trans*-cinnamic acid at 290 nm. Thus, 50 μL of cell lysate was added to the solution of *p*-methoxyphenylalanine (2 mM final concentration) in 50 mM Tris.HCl, 100 mM NaCl pH = 8.8, yielding a final reaction volume of 200 μL. The enzyme activity was assessed by monitoring the absorbance at 290 nm over 15 min, at 30 °C, using a Tecan Infinite Spark 10M plate reader and UV-transparent polyacrylate 96-well plate (Costar #3635, Corning, Waltham, MA, USA) The DNA of the hits with similar or increased enzyme activity, compared to I460V-*Pc*PAL, was extracted and sequenced (BIOMI, Gödöllő, Hungary) to identify the mutations.

### 2.3. Protein Expression, Isolation, and Thermal Unfolding Assessments

Wild-type *Pc*PAL and variants I460S, I460T, and I460V were expressed, isolated, and purified using our previously reported method [14,35]. The protein homogeneity/oligomerization state was determined through size-exclusion chromatography using Superdex 10/300 (GE Healthcare Bio-Sciences, Uppsala, Sweden), while the purity of the enzymes was assessed by SDS-PAGE. The thermal unfolding of the *Pc*PAL mutants was determined by nanoscale differential scanning fluorimetry measurements, using Prometheus NT.48 nanoDSF instrument (NanoTemper Technologies, München, Germany).

### 2.4. Enzyme Kinetics

The kinetic measurements were performed by monitoring the production of *trans*-cinnamic acid and/or *p*-methoxy *trans*-cinnamic acid at 290 nm, using substrate concentrations of 50–5000 μM and purified *Pc*PAL variants at a fixed enzyme concentration of 0.1543 μM. The v_max_, K_M_, and k_cat_ values were obtained from the non-linear regression fitting of the Michaelis–Menten curves.

### 2.5. HPLC Analysis for Conversion and Enantioselectivity Determinations

The reactions with whole cell biocatalysts were performed in 20 mM Tris.HCl, 100 mM NaCl pH = 8.8 using 2 mM substrate concentration and induced whole cells of a cell density of OD_600_ ~2 (corresponding to a wet cell concentration of ~12 mg/mL) in a final volume of 200 μL. In case of reactions using purified enzymes as biocatalysts, similar conditions were employed using 50 μg purified *Pc*PAL variant. The reaction mixtures were incubated at 800 rpm, 30 °C, taking samples after 2, 4, 6, and 16 h reaction times. Samples were quenched by adding an equal volume of MeOH and centrifugated at 13,400 rpm (12,000× *g*) for 10 min, filtered through a 0.22 μm nylon membrane filter and analyzed by HPLC. In order to determine the conversions values, a Gemini NX-C18 column (150 × 4.5 mm; 5 µm) was chosen, using as mobile phase: A: NH_4_OH buffer (0.1 M, pH 9.0)/ B: MeOH, with a flow rate of 1.0 mL/min. The enantiomeric excess was determined by chiral HPLC separation, using Crownpak CR-I (+) chiral column (150 × 3 mm; 5 µm) and HClO_4_ (pH = 1.5)/acetonitrile as mobile phase at a flow rate of 0.4 mL/min. All measurements were performed at 25 °C.

### 2.6. Analyzing Thermal Unfolding of PcPAL Mutants through nanoDSF Measurements

Nanoscale differential scanning fluorimetry (nanoDSF) measurements were performed using Prometheus NT.48 nanoDSF instrument, which monitors the shift of intrinsic tryptophan fluorescence of proteins upon unfolding by detecting the fluorescence at emission wavelengths of 330 and 350 nm. For determination of the protein melting point (T_m_), the maximum of the first derivative of the fluorescence ratios F350/F330 was used.

*Pc*PAL variants were diluted with 50 mM Tris.HCl, 100 mM NaCl pH 8.8 buffer to a final concentration of 1 mg/mL. 10 μL of each sample was loaded into UV capillaries (NanoTemper Technologies, München, Germany) and protein unfolding was detected during heating in a linear thermal ramp of 1 °C/min between 20 and 95 °C, with an excitation power of 20%. Data analysis was performed using NT Melting Control software and melting temperature (T_m_) was determined by fitting the experimental data using a polynomial function, in which the maximum slope was indicated by the peak of its first derivative (F350/F330). All measurements were performed in triplicate.

### 2.7. Molecular Docking

The enzyme active site was specified from the coordinates of the co-crystallized ligand (*E*)-3-(4-methoxyphenyl)acrylic acid within the crystal structure of *Pc*PAL I460V (PDB ID: 6RGS). A docking grid with a size of 16 Å × 16 Å × 16 Å was used, and the grid center was obtained from the coordinates of the co-crystallized ligand from 6RGS. Ligand docking was performed using Autodock Vina [36], with the degree of exhaustiveness set to 200. Residues V460, T460, and I460 were explicitly specified as flexible side-chains in mutant I460V, I460T, and wild-type *Pc*PAL, respectively. Protonation states of the protein scaffold were predicted at pH 8.8 and 10 by the PROPKA method using PDB2PQR [37].

The ground state geometries of substrates *p*-MeO-Phe and *p*-MeO-cinnamic acid were obtained at the DFT level of theory, employing the B3LYP functional and the 6–311++G(d,p) basis set, using the Gaussian 09 package. Harmonic vibrational frequencies have been checked for the optimized structures to confirm that the true energy minimum has been found.

## 3. Results and Discussion

### 3.1. Site-Saturation Mutagenesis

In the initial randomization library for I460, the commonly employed NNK degeneracy, defining 32 codons encoding all 20 canonical amino acids and one Stop codon, was applied. For the generation of the mutant libraries, two different mutagenesis strategies were employed, one based on partially overlapping primers [38] and the Megaprimers method [39].

The procedure using partially overlapping primers is known for its increased efficiency in comparison with the QuikChange procedure and employs primer pairs containing non-overlapping sequences at their 3′ end and mutagenic, primer-primer complementary (overlapping) sequences at the 5′ end [30,31]. The melting point of the non-overlapping region (T_m no_) positions 5 to 10 °C higher than those of primer-primer complementary region (T_m pp_), in order to avoid the competing self-pairing. For a high-quality DNA library, two primer pairs (Table S1), one favoring the annealing of the codons rich in bases G, C, and one favoring those based predominantly on A or T, were employed. Thus, the products of the two PCR reactions, performed at different annealing temperatures, were combined, digested with DpnI and transformed into *E. coli* BL21 (DE3) pLys competent cells through heat shock and electroporation.

**Figure 1:**
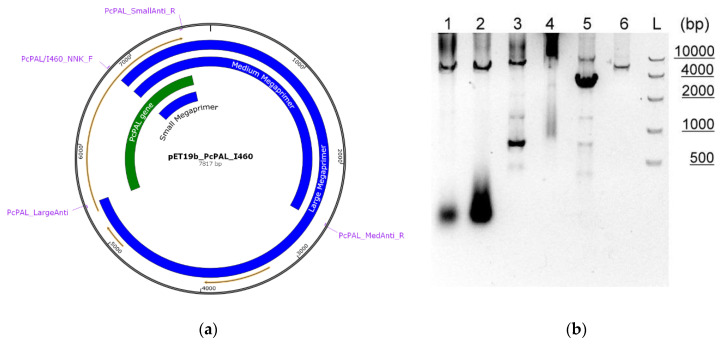
(**a**) Saturation mutagenesis using the Megaprimers procedure, representing the gene encoding *Pc*PAL (*green*), the pET19b-vector backbone (*black*), and the megaprimers of the different, small, medium, large lengths (*blue*). In the first PCR stage, the forward mutagenic primer and the reverse, antiprimer anneal to the template resulting the amplified sequences of the corresponding, large, medium, and small-sized megaprimers. In the second PCR stage through annealing of the forward primer and the obtained megaprimers, the mutated plasmid products are obtained. (**b**) Agarose gel analysis of the PCR products obtained through the different mutagenesis procedures: *lanes* 1, 2 using the two sets of partially overlapping primers, *lane* 3 small-megaprimer protocol, *lane* 4 large-megaprimer protocol, *lane* 5 medium-megaprimer protocol, *lane* 6 optimized small-megaprimer protocol, *L* 10 kb DNA ladder.

In the case of the megaprimer procedure [25,32,33], a forward mutagenic primer carrying the NNK degeneracy and a reverse non-mutagenic primer were used in a two-stage PCR reaction. Reverse primers for megaprimers of the different, small, medium, and large lengths were designed (Figure 1a, Table S1).

While by the use of large megaprimer (Figure 1b lane 4), no plasmid amplification could be observed, the medium-sized megaprimer procedure (Figure 1b lane 5) provided low plasmid concentration within the second PCR step compared to the megaprimer concentration resulted from the first PCR step. Comparing the concentration of the desired plasmid product with that of the megaprimer product, it was found that the use of small-sized megaprimers (Figure 1b lane 3) competed with the partially overlapping primer procedure (Figure 1b lanes 1 and 2). Accordingly, further optimization steps of the PCR protocol, related to the template concentration and extension temperature were performed, significantly increasing the quality of the PCR product of the small-megaprimer protocol (Figure 1b lane 6).

### 3.2. Quality of Transformant Libraries

**Figure 2:**
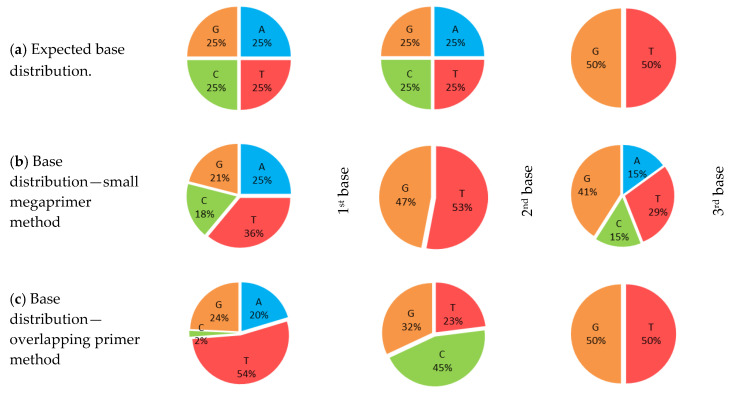
Nucleotide-base distribution within randomized position 460 of *Pc*PAL. (**a**) Theoretical, expected distribution for NNK-degeneracy, with the four bases (A, T, G, C) equally represented in the first two nucleotides, while G, T having the same ratio within the third position); (**b**) experimental base-distribution from the small megaprimer-transformant library; (**c**) experimental base-distribution from the transformant library obtained by the partially overlapping primers mutational strategy. For both (**a**) and (**c**) cases, the base-distribution was assessed by sequencing the DNA plasmid isolated from the cellular mixture of all colonies pooled from the LB-agar plate of the corresponding transformant library. The G, T, C, A encodes for the corresponding nucleotide bases thymine (T), adenosine (A), cytosine (C), and guanine (G).

Directed evolution procedures, such as (iterative) saturation mutagenesis, require a high-efficiency transformation of the mutagenesis product, providing a sufficient number of colonies for the 95% coverage of residue randomization, 96 colonies in case of a single-site NNK degeneracy. In order to generate the requested high colony number both heat shock and electroporation transformation of the PCR products obtained through the partially overlapping primers strategy and by the optimized small-sized megaprimer strategy were tested using BL21 (DE3) pLys, Rosetta (DE3) pLys, or BL21 (DE3) Gold strains *E. coli* competent cells. While by heat-shock, low transformation efficiency (<10 colonies) was obtained for both PCR products for each host strains, the electroporation protocol provided <31 colonies in case of *E. coli* BL21 (DE3) pLys, Rossetta (DE3) pLys and >140 colonies in case of BL21 (DE3) Gold host. Further, to evaluate the diversity and quality of libraries obtained by the partially overlapping primers and the small-sized megaprimer mutagenesis methods, we compared the distribution of the encountered bases at the degeneracy site for the plasmids isolated from the pooled agar plate of the two transformant libraries with the values expected of NNK-degeneracy (Figure 2, Figure S1). While the transformant library generated by the small-megaprimer mutational strategy resembled the theoretical base distribution, in case of the partially overlapping primer strategy-derived library, within the first and second base of the NNK-degeneracy, cytosine (C) was underrepresented or completely missing, with a high dominance of thymine (T) within the first degenerated nucleotide. Accordingly, from the two transformant libraries, only the higher-quality one generated by the small megaprimer-strategy limits the occurrence of amino acid bias and ensures that all the desired mutations are present within the transformant library. The differences in the distribution percentages for the different bases, obtained for the small megaprimer-transformant library, with respect to the theoretical one, is similar to those observed in previous saturation mutagenesis studies [40] and approaches the limits of saturation mutagenesis procedures [41].

### 3.3. Screening of Transformant Libraries

#### 3.3.1. Assay Development

**Figure 3:**
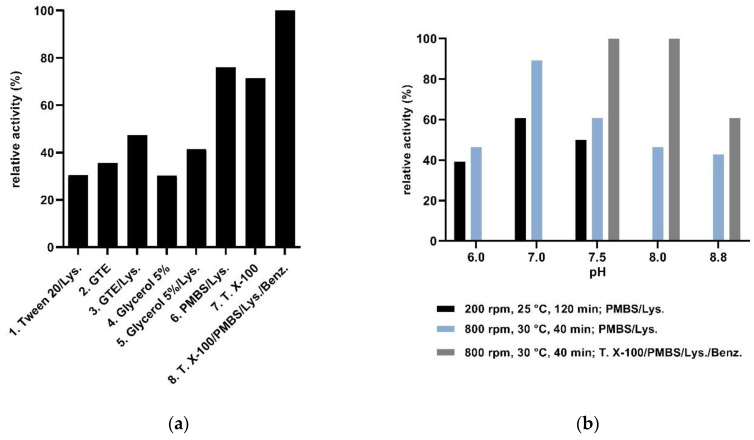
Assay optimizations monitoring the relative activity of the different cell lysates of I460V-*Pc*PAL, in the ammonia elimination reaction of *p*-MeO-Phe. The following lysis buffers have been employed: (**a**) 1. Tween 20 buffer (50 mM NaH_2_PO_4_·2H_2_O, 300 mM NaCl, 10 mM imidazole, 0.07 mM lysozyme, 400 U/mL DNAse I. 1% Tween20 pH 8), 2. GTE buffer (50 mM glucose, 10 mM EDTA, 25 mM Tris.HCl, pH 8), 3. GTE/Lys. buffer (50 mM glucose, 10 mM EDTA, 0.07 mM lysozyme, 25 mM Tris.HCl, pH 8), 4. 5% glycerol buffer (5 % glycerol, 100 mM NaCl, 1 mM DTT, 50 mM Tris.HCl, pH 7,5), 5. 5% glycerol/Lys. buffer (5 % glycerol, 100 mM NaCl, 1 mM DTT, 0.07 mM lysozyme, 50 mM Tris.HCl, pH 7,5), 6. PMBS/Lys. buffer (0.36 mM PMBS, 0.07 mM lysozyme, 50 mM NaCl, 20 mM Tris.HCl, pH 8.8), 7. Triton X-100 buffer (2% Triton X-100, 50 mM NaCl, 20 mM Tris.HCl, pH 8.8), 8. Triton X-100/PMBS/Lys./Benz. buffer (2% Triton X-100, 0.36 mM PMBS, 0.07 mM lysozyme, 25 U/mL benzonase, 50 mM NaCl, 20 mM Tris.HCl, pH 8.8). (**b**) Optimizations using the two component PMBS/Lys. buffer and the four-component Triton X-100/PMBS/Lys./Benz. buffer systems with different pHs and/or lysis conditions. Abbreviations: Lys., PMBS, T. X-100 and Benz. denote for lysozyme, polymyxin B sulphate, Triton X-100 and benzonase, correspondingly.

Since saturation mutagenesis-based enzyme engineering requires a reliable high-throughput activity assay able to screen the large transformant libraries, the compatibility of the UV spectroscopy-based PAL-activity assay [26,42] with the use of whole cell PAL-biocatalysts was tested. Unfortunately, the low sensitivity, derived from the high UV-background of cell suspensions hindered the detection of mutant variants with still low, but increased activity compared to the *wild-type* enzyme, that would serve as potential hits and starting points for additional saturation mutagenesis rounds. Therefore, the UV-assay—based on monitoring the production or consumption of cinnamic acid derivatives—was appropriately adapted for reliable enzyme activity measurements using cell lysates as biocatalysts (Figure 3a,b). For cell lysis, various known lysis-inducing chemical agents, such as Tween-20, Triton-X-100, lysozyme, polymyxin B sulphate, glucose, and glycerol solutions were employed comparing their efficiency by activity measurement of cell lysates within the natural PAL-reaction.

Cell lysates obtained by the use of Triton X-100 or polymyxin B sulfate (PMBS) or their combined use with lysozyme showed significantly higher specific activity compared to those obtained by Tween-20, glucose, or glycerol-based solutions (Figure 3a). Next, the optimization of the most efficient cell lysis procedures was performed. A combination of the three efficient cell-lysis agents, Triton-X, PMBS, and lysozyme, significantly increased the viscosity of the cell lysate, hindering the appropriate purification of cell lysate by centrifugation, providing as a consequence a high standard deviation within the activity assay. The additional use of benzonase provided a four-component cell lysis cocktail with increased efficiency (Figure 3a, lane 8). However, in high-throughput experiments, this method was improper due to the high price of benzonase. Therefore, the two-component PMBS-lysozyme lysis buffer was selected, and its efficiency was further improved by optimizing the pH, incubation conditions (temperature, rpm) (Figure 3b), thus finally the cell lysis performed for 40 min at 800 rpm, 30 °C with lysis buffer containing 0.36 mM of PMBS and 0.07 mM lysozyme, pH 7.0, provided homogenous, clear cell lysate with low UV-background, applicable for the high-throughput PAL-activity screens (Figure 3b, lane 4, Table S3).

#### 3.3.2. Activity Screens of Transformant Libraries

As previously shown [14,16], mutation I460V confers activity to *Pc*PAL towards several phenylalanine analogs, such as *p*-methoxyphenylalanine, poorly or not accepted as substrates by the *wild-type* enzyme. In order to identify other possible mutants with significantly increased activity, the saturation mutagenesis libraries obtained from randomization at residue 460 of *Pc*PAL were tested in a high-throughput format for the ammonia elimination from *p*-MeO-Phe using the optimized activity assay.

The activity of >100 colonies/saturation library, providing >95% coverage for the used NNK randomization, was screened, of which 10 hits displayed similar or higher enzyme activity than the cell lysate of the highly active I460V *Pc*PAL, used as a positive control. Among the hits, besides the I460V mutation identified in 4 selected colonies, 5 novel active mutants, I460C-, I460L-, I460W-, I460T-, I460S-*Pc*PAL were also revealed.

**Figure 4:**
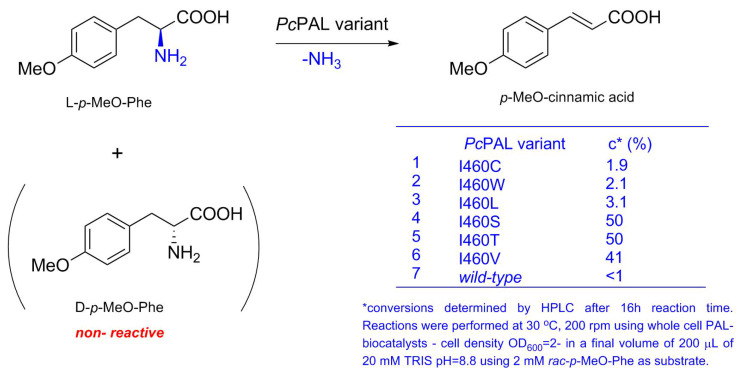
Ammonia elimination from racemic *p*-MeO-Phe using the induced whole-cells of the corresponding *Pc*PAL variants showing increased activity within the high-throughput activity screens.

To confirm the increased enzyme activity of the identified mutants, the conversion values of the ammonia elimination from racemic *p*-MeO-Phe using the corresponding *Pc*PAL variants as whole cell-biocatalysts were determined by HPLC (Figure 4). While variants I460C, I460L, and I460W showed low conversions after 16 h reaction time, both variants I460T and I460S showed high conversions, exceeding those obtained by the positive control I460V (Figure 4, Figures S3–S5) and approaching the maximal, 50% conversion values of a kinetic resolution process.

### 3.4. Functional and Structural Characterization of Improved PcPAL Variants

Further, mutants I460S, I460T of enhanced activity and I460V, as reference were isolated and the ammonia eliminations from *p*-MeO-Phe were performed using the purified enzymes as biocatalysts.

**Figure 5:**
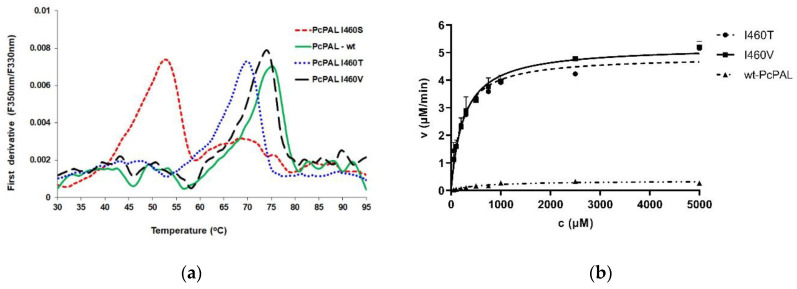
(**a**) The thermal unfolding temperature (T_m_) of I460S *Pc*PAL and I460T *Pc*PAL, determined by nanoDSF (Prometheus NT.48). Fluorescence intensity ratios F350/F330 and their first derivative are represented as a function of the applied linear thermal ramp (**b**) Michaelis–Menten curves obtained by initial reaction velocity measurements using *p*-MeO-Phe as substrate at different concentration and purified *Pc*PAL variants as catalysts.

While both I460V and I460T showed conversions approaching the maximal 50% conversion of the kinetic resolution process after only 2 h reaction time, I460S provided significantly lower, 17% conversion, approaching the maximal value only after 16 h (Figure S10). The significant activity discrepancy between the free and intracellular enzyme suggested stability issues for variant I460S, which was supported by the thermal unfolding profiles analysis of all active mutants. As depicted in Figure 5a, the melting temperature (T_m_) of variant I460S (55 ± 0.2 °C) was significantly lower compared to the *wt-Pc*PAL (75.1 ± 0.2 °C) or to the variant I460T (70 ± 0.2 °C), supporting a decrease of the protein stability induced by the mutation. A similar effect was also reported for the rationally designed I460A *Pc*PAL mutant [16] with decreased stability and consequently, low enzyme activity of the purified enzyme.

In case of reactions performed with purified enzymes, besides the conversions, the enantiomeric excess (ee) of the unreacted d-*p*-MeO-Phe was also monitored by chiral HPLC. Both I460S and I460T-catalyzed reactions resulted in optically pure (ee >99%) d-*p*-MeO-Phe (Figures S11 and S12), at 50% conversions, equal to the theoretical maximum of a kinetic resolution process, which supports the complete enantioselectivity of variant I460S and I460T within the model reaction.

Further, the kinetic parameters of *Pc*PAL variants I460T, I460V were determined within the ammonia elimination of *p*-MeO-Phe (Figure 5b, Table 1) and of the natural substrate l-phenylalanine (l-Phe) (Figures S14–S16, Table 1). Both I460V- and I460T-*Pc*PAL showed similar catalytic efficiency within the ammonia elimination from the non-natural substrate *p*-MeO-Phe, while the wild-type *Pc*PAL poorly catalyzed this reaction. Despite of the ~10 fold lower k_cat_ values shown by the mutants within the ammonia elimination from *p*-MeO-Phe than that of the *wild-type-Pc*PAL in the natural, l-Phe deamination reaction (Table 1), these mutants could be considered potent biocatalysts, since PALs with similar catalytic efficiencies were employed for efficient preparative biotransformations of several phenylalanine analogs [14,16,43]. Interestingly, variant I460T exhibits significantly higher affinity towards the natural substrate, in comparison to reference mutant I460V, thus also preserving the specificity order shown by the *wild-type Pc*PAL.

### 3.5. Molecular Docking

**Figure 6:**
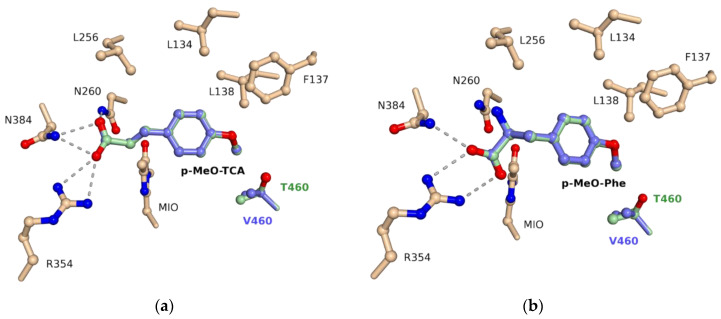
View of the catalytic site, including the electrophilic 4-methylideneimidazol-5-one (MIO) prosthetic group; overlay of the (**a**) *p*-MeO cinnamic acid and (**b**) *p*-MeO-Phe orientations within the catalytic site of I460V and I460T.

Based on the recently reported crystal structure of variant I460V of *Pc*PAL in complex with *p*-methoxy-*trans*-cinnamic acid (PDB ID: 6RGS) [14], we performed molecular docking of the l-*p*-methoxyphenylalanine and the corresponding cinnamic acid in case of variant I460T, reference mutant I460V and *wild*-*type Pc*PAL (Figure 6). The provided orientation of *p*-MeO-cinnamic acid, similar to one observed within the crystal structure (Figure S20), supported the validity of the docking procedure, which supports almost identical, favorable substrate orientations within the catalytic site of both I460V and I460T variants. Moreover, the docking results confirmed the inactivity of the *wild*-*type Pc*PAL. In this case, no active conformation for *p*-MeO-Phe or of its cinnamic acid counterpart could be observed, due to the steric repulsion with residue I460 (Figure S21).

## 4. Conclusions

This study focuses on the saturation mutagenesis of activity modulator residue I460 of *Pc*PAL in order to explore and identify novel PAL-variants with increased activity towards non-natural substrates. Using the NNK-degeneracy, providing access to all possible single-site mutants, the optimized mutagenesis experiments provided high-quality mutant libraries, with nucleotide-base distributions approaching the theoretical values. For the activity screens of mutant libraries, a high-throughput PAL-activity assay was adapted for use with cell-lysate biocatalysts. During the activity screen for the ammonia elimination reaction of the non-natural substrate, *p*-methoxyphenylalanine, several hits have been identified, showing similar or higher activity than the cell lysate of the rationally developed, highly active I460V *Pc*PAL. Interestingly, among the hits the mutation I460V occurred with the highest frequency, supporting the efficiency of the reported rational design approach [14,16]. Further biotransformations using the whole-cell and/or purified PAL-biocatalysts of the corresponding mutants confirmed the activity of the novel variants towards *p*-MeO-Phe, poorly transformed by the *wild-type* enzyme. While within the whole-cell biotransformations, variants I460T and I460S presented an improved catalytic efficiency compared to the rationally explored I460V *Pc*PAL, mutant I460S presented stability issues and low catalytic efficiency after purification. Variant I460T, in the model reaction with *p*-MeO-Phe, showed similar kinetic profile with the reference variant I460V, moreover, in contrast to I460V, maintained the catalytic efficiency of the *wild-type* enzyme within the natural reaction. Molecular docking based on the recently reported crystal structure of I460V *Pc*PAL (6RGS) complexed with the product of the investigated model reaction, *p*-MeO-cinnamic acid, supported similar substrate orientations within the active site of variants I460V and I460T, while no active orientation of the substrate could be observed in the case of the *wild-type Pc*PAL. The developed/optimized procedures provide tools for efficient saturation mutagenesis based directed evolution of PALs, while the identified variants, due to the highly conserved nature of residue I460V among phenylalanine-ammonia lyases provide further perspective for PAL mediated biotransformations of industrially relevant phenylalanine analogs.

## Figures and Tables

**Table 1:** Enzyme kinetic parameters for the ammonia eliminations from *p*-methoxyphenylalanine and natural substrate l-Phe catalyzed by *wild-type Pc*PAL and variants I460.

Compound	*4-MeO-Phe*			*l-Phe*		
*Pc*PAL	*wt-*	I460T	I460V	*wt-*	I460T	I460V
**K_M_ (μM)**	1741 ± 191.2	210.2 ± 27.9	256.2 ± 26.8	110.9 ± 12.3	127.1 ± 20.7	474.6 ± 39.8
**k_cat_ (s^−1^)**	0.012	0.129	0.139	0.698	0.805	0.617
**v_max_ (μM/min)**	0.45 ± 0.03	4.8 ± 0.1	5.22 ± 0.1	26.2 ± 0.6	35.4 ± 1.1	5.2 ± 0.1
k_cat_/ K_M_ *10^−3^ **(s^−1^xμM^−1^)**	0.007	0.617	0.543	6.297	6.334	1.300

## Supplementary Materials

The following are available online at https://www.mdpi.com/2218-273X/10/6/838/s1: Supporting Information describing experimental details/results for saturation mutagenesis, assay development, HPLC reaction monitoring, enzyme kinetics, enzyme purification and molecular docking.
