# Supplementary material for: Saturation Mutagenesis for Phenylalanine Ammonia Lyases of Enhanced Catalytic Properties
Source: Biomolecules. 2020 May 30;10(6):838. doi: 10.3390/biom10060838 (PMC7355458; doi:10.3390/biom10060838)
Supplement: Supplementary file 1 [file biomolecules-10-00838-s001.pdf]

biomolecules MDPI

Supporting Information

Saturation Mutagenesis for Phenylalanine Ammonia Lyases of Enhanced Catalytic Properties

Raluca Bianca Tomoiagă, Souad Diana Tork, Ilka Horváth, Alina Filip, Levente Csaba Nagy and László Csaba Bencze*

Biocatalysis and Biotransformation Research Center, Faculty of Chemistry and Chemical Engineering, Babeș-Bolyai University, Arany János Str. 11, RO-400028 Cluj-Napoca, Romania
* Correspondence: cslbencze@chem.ubbcluj.ro

Table Sof Contents

1. Materials 2

2. Instrumentation 2

3. Site-Saturation mutagenesis....................2

3.1 *ISM Primers*....................2

3.2 *Estimation of Parental Content from the Sequencing Chromatogram of the PCR Products* 3

4. Assay Optimization....................4

5. HPLC Monitoring of the Enzymatic Reactions 4

5.1. *Determination of Conversion Values by HPLC*....................4

5.2 *Biotransformations*....................4

5.3 *Representative Chromatograms.* 5

5.4 *HPLC Method to Determine the Enantiomeric Excess (ee) of p-Methoxy-rac-Phenylalanine*....................8

6. SDS-PAGE Gel From the Purification Steps of I460T-*Pc*PAL and I460S-*Pc*PAL....................11

7. Enzyme Kinetics 11

8. Molecular Docking 14

1. Materials

The commercial chemicals and solvents were products of Sigma Aldrich or Alfa-Aesar. The primers used for the mutagenesis were purchased through services of Genomed (Debrecen, Hungary). IPTG, Phusion Hot Start DNA Polymerase, dNTPs, DpnI, XhoI, Bpu1102I, agarose were all products of Thermo Fischer Scientific (Waltham, MA, USA). Plasmid extraction kit and ethidium bromide were purchased from Sigma-Aldrich (St. Louis, MO, USA). LB medium was from Liofilchem (Roseto, Italy), protease inhibitor cocktail from Hoffman La-Roche (Basel, Switzerland), while the Ni-NTA Superflow resin used for affinity chromatography was from Qiagen (Hilden, Germany).

2. Instrumentation

The differential scanning fluorimetry (nanoDSF) measurements were performed using Prometheus NT.48 instrument (NanoTemper Technologies, Munich, Germany). High performance liquid chromatography (HPLC) analyses were conducted with Agilent (Santa Clara, CA, USA) 1200, 1260 and 1100 systems. Kinetic measurements were performed on TECAN Spark 10M equipped with a TE-cool module. Mastercyler proS from Eppendorf (Hamburg, Germany) was used to perform the PCR reactions, while the electroporation was carried out using 1 mm cuvettes and an Eppendorf electroporator (Hamburg, Germany). Gene sequencing services were performed through BIOMI Ltd. (Gödöllő, Hungary).

3. Site-Saturation mutagenesis

3.1. ISM Primers

The *Pc*PAL mutants were obtained through site-saturation mutagenesis. The primers containing the NNK degenerate codons were synthesized by Invitrogen (Germany) and are listed in Table S1 and S2.

Table S1. List of the primers used for saturation mutagenesis.

Primer name	Sequence	T_m (^{0}C) (min-max)	$T_{m\ no}$	$T_{m\ pp}$	Base Pair
PcPAL-I460_NNK_F	AAGGGAGCCGAA*NNK*GCCATGGCCTCC	64.3-68.8	-	-	27
PcPAL_SmallAnti_R	GCTCAGCTCAGGAAATGGGCAAGGGGG	65.8	-	-	27
PcPAL_MedAnti_R	CTCTGGTCCCGCCGCATCCATACCG	65.9	-	-	25
PcPAL_LargeAnti_R	GTGGCTCCGTTTCCGTTTTCCATCTCGAG C	65.7	-	-	30
PcPAL_I460_FOR1	GCCGAA*NNK*GCCATGGCCTCCTACTGCT CC	67-71	66	57-61	30
PcPAL_I460_REV1	ATGGC*MNN*TTCGGCTCCCTTGAATCCGT AGTCCAA	65-69	66	57-61	35
PcPAL_I460_FOR2	GCCGAA*NNK*GCCATGGCCTCCTACTGCT CCGAATTGC	70-73	62-67	73	37
PcPAL_I460_REV2	ATGGC*MNN*TTCGGCTCCCTTGAATCCGT AGTCCAAGGAGG	69-73	62-67	72	40

Table S2. Saturation mutagenesis of I460 pET19b_pcpal using two protocols.

Protocol	Primer combination	Megaprimer length
**Megaprimer**		
Small	PcPAL-I460_NNK_F/PcPAL_SmallAnti_R	794
Medium	PcPAL-I460_NNK_F/PcPAL_MedAnti_R	3667
Large	PcPAL-I460_NNK_F/PcPAL_LargeAnti_R	6476
**Partially overlapping primers**		
PCR1	PcPAL_I460_FOR1/PcPAL_I460_REV1	-
PCR2	PcPAL_I460_FOR2/PcPAL_I460_REV2	-

3.2 Estimation of Parental Content From the Sequencing Chromatogram of the PCR Products

Peak intensities of the four different chromophores representing adenine (A), cytosine (C), guanidine (G) and thymine (T) from each sequencing result were evaluated with Chromas Lite and represented in pie diagrams (Figure S1) followed by their comparison to the theoretical NNK randomization of position 460 in pET19b_pcpal.

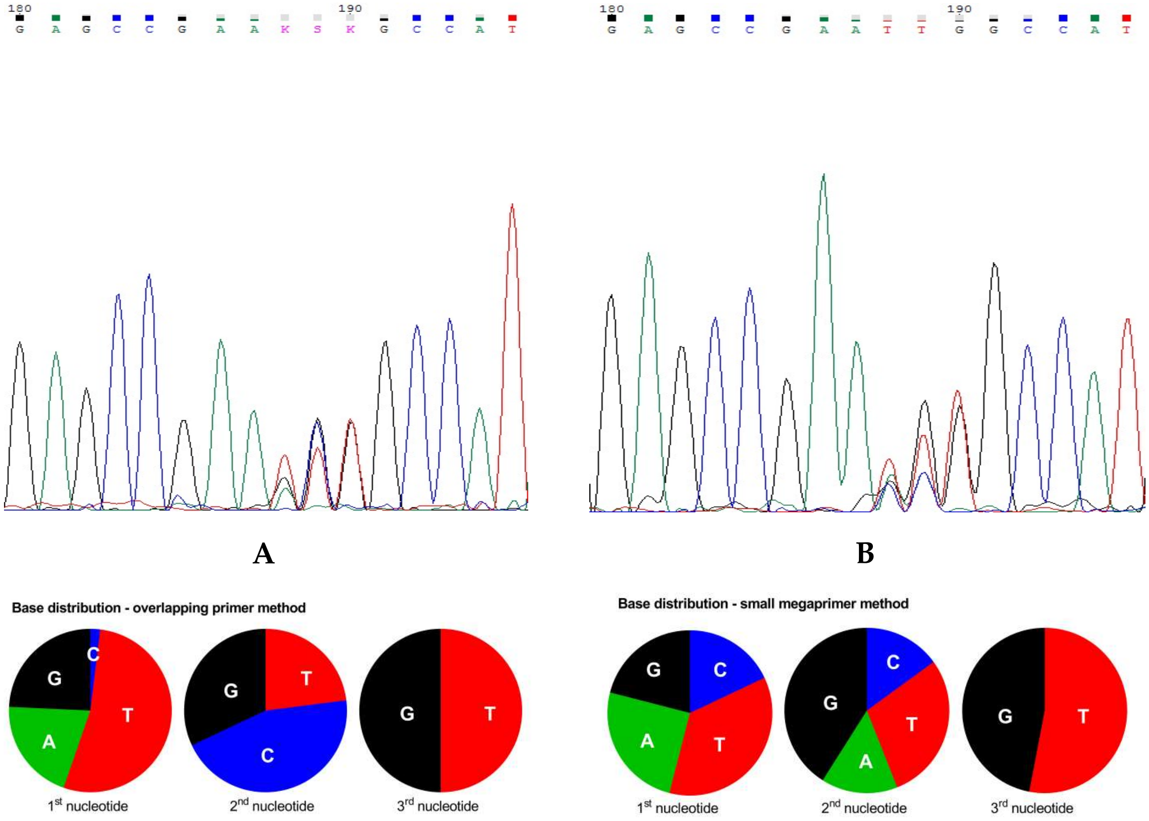

Figure S1. Sequencing chromatogram and base distribution for colony pooling after the transformation of the PCR product using (**A**) partially overlapping primers (**B**) small megaprimer.

4. Assay Optimization

The activity of I460V *Pc*PAL was assessed at different reaction conditions, using 0.07 mM lysozyme and 0.36 mM PMBS in PBS buffer (20 mM PBS, 50 mM NaCl). The measurements were performed using 100 μL of 4 mM 4-MeO-Phe in TRIS buffer (50 mM Tris.HCl, 100 mM NaCl, pH 8.8), 50 μL lysate, in 200 μL reaction volume, following the formation of the (*E*)-3-(*p*-methoxyphenyl)-acrylic acid at 290 nm.

Table S3. The activity of I460V *Pc*PAL at different reaction conditions.

Reaction Conditions	Activity (OD_{290}/h)		
	**pH = 6**	**pH = 7**	**pH = 7.5**
800 rpm, 30 °C, 40 min	0.132	0.255	0.175
200 rpm, room temperature, 2h	0.115	0.177	0.156

* all measurements were performed in triplicate.

5. HPLC Monitoring of the Enzymatic Reactions

5.1. Determination of Conversion Values by HPLC

The conversions of the *Pc*PAL-catalyzed ammonia elimination reactions were calculated using the relative response factor of the 4-MeO-cinnamic acid compared to 4-MeO-Phe, that was determined by injecting the mixture of known composition of the racemic 4-methoxy-phenylalanine and the 4-MeO-cinnamic acid onto Gemini NX-C18 column (150 × 4.5 mm; 5 μm).

Table S4. HPLC method and response factor used for the conversion value determinations.

Compound	Eluent* [% B]	Retention Time (min)		Response Factor	Wavelength Used for UV Detection
		***rac*-4-MeO-Phe**	**4-MeO cinnamic acid**	***rac*-4-MeO-Phe *vs* 4-MeO-cinnamic acid**	
rac-4-MeO-phenylalanine and 4-MeO-cinnamic acid	25 to 32 in 8 min	3.1	6.1	0.622	220

*Mobile phase: A: NH_4OH buffer (0.1 M, pH 9.0) / B: MeOH; flow rate: 1.0 mL min^{-1}, measurements performed at 25 °C, using Gemini NX-C18 column (150 × 4.5 mm; 5 μm).

5.2. Biotransformations

Table S5. HPLC conversions of the ammonia elimination from *rac*-4-methoxy-phenylalanine after 16 h, using induced whole cell harbouring the plasmids of the corresponding *Pc*PAL variants.

***Pc*PAL variant**	**Conversion (%)**
I460C	1.9
I460W	2.1
I460L	3.1
I460T	~50
I460S	~50
I460V	41

Table S6. HPLC conversions of the ammonia eliminations from *rac*-4-methoxy-phenylalanine using purified *Pc*PAL variants I460V, I460T, I460S as biocatalysts.

***Pc*PAL**	**Conversion (%)**			
**Variant**	**2 h**	**4 h**	**6 h**	**16 h**
I460V	47	~50	~50	~50
I460T	47	~50	~50	~50
I460S	17	28	35	43

5.3. Representative Chromatograms.

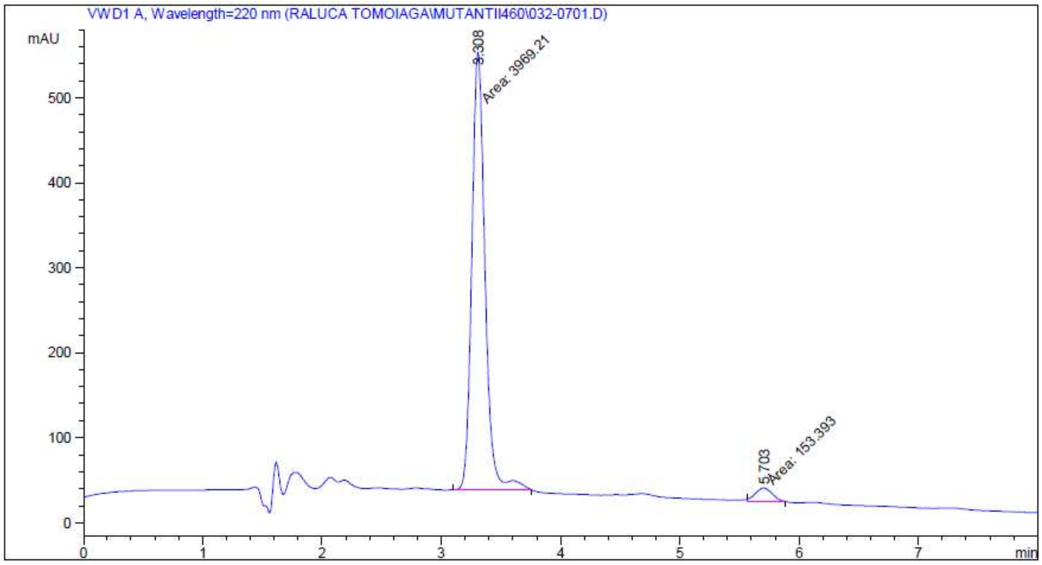

Figure S2. HPLC chromatogram from Gemini NX-C18 column of the whole cells I460W-*Pc*PAL catalyzed ammonia elimination reaction of *rac*-4-methoxy-phenylalanine after 16 h, with the specific signals of *rac*-4-methoxy-phenylalanine (3.3 min) and of the produced 4-MeO-cinnamic acid (5.7 min).

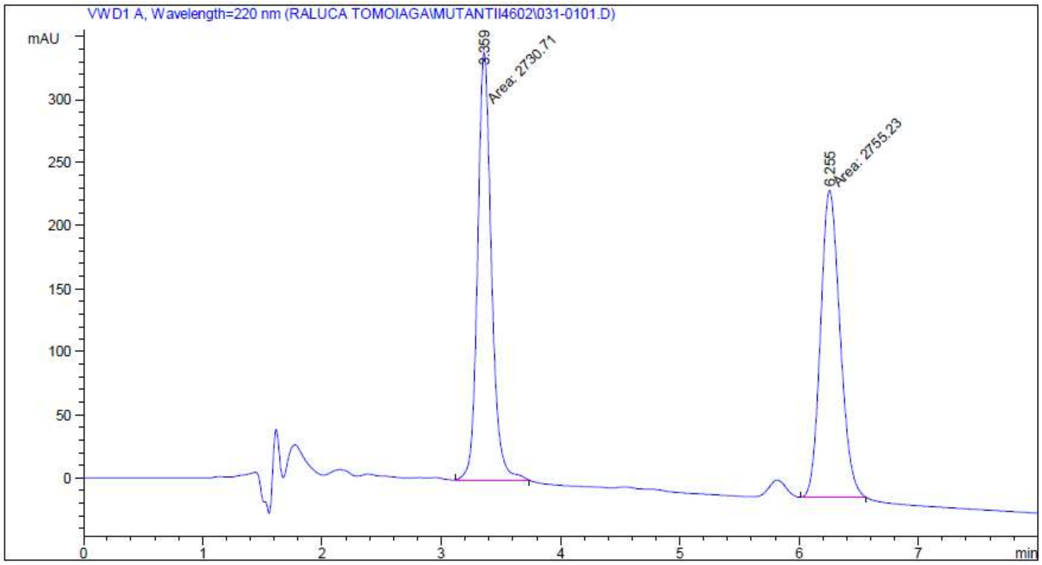

Figure S3. HPLC chromatogram from Gemini NX-C18 column of the whole cells I460V-*Pc*PAL catalyzed ammonia elimination reaction of *rac*-4-methoxy-phenylalanine after 16 h, with the specific signals of *rac*-4-methoxy-phenylalanine (3.3 min) and of the produced 4-MeO-cinnamic acid (6.2 min).

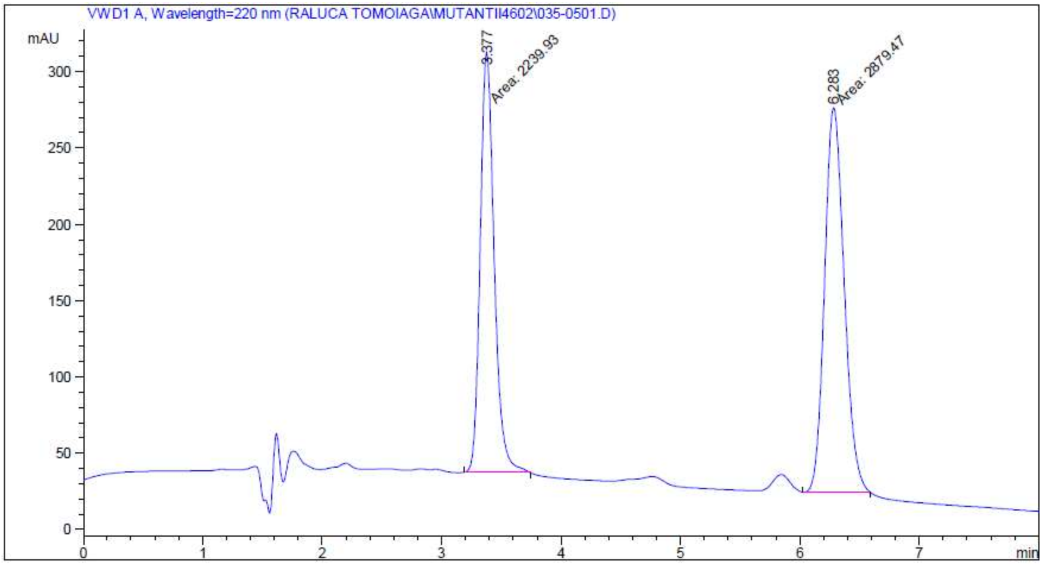

Figure S4. HPLC chromatogram from Gemini NX-C18 column of the whole cells I460T-*Pc*PAL catalyzed ammonia elimination reaction of *rac*-4-methoxy-phenylalanine after 16 h, with the specific signals of *rac*-4-methoxy-phenylalanine (3.3 min) and of the produced 4-MeO-cinnamic acid (6.2 min).

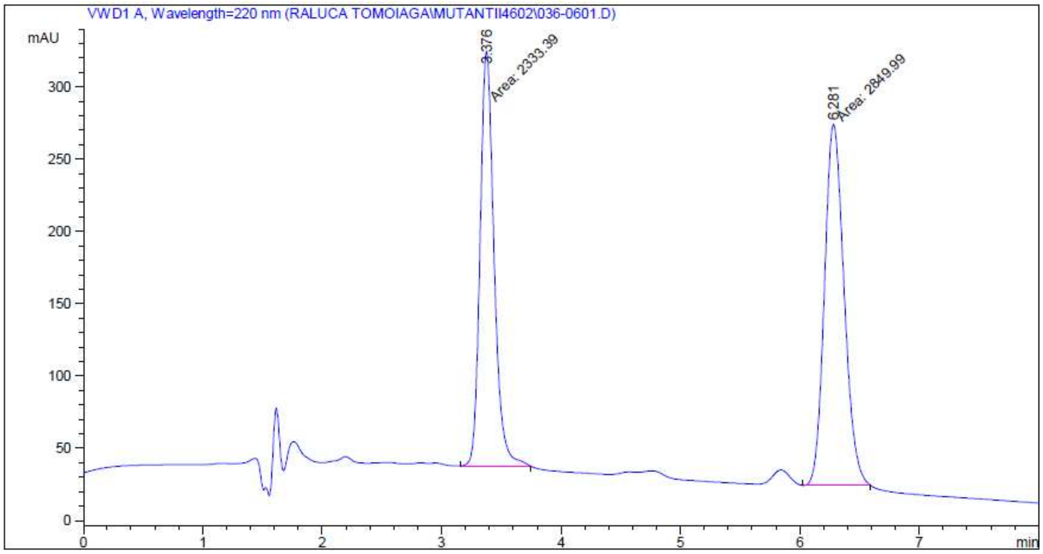

Figure S5. HPLC chromatogram from Gemini NX-C18 column of the whole cells I460S-*Pc*PAL catalyzed ammonia elimination reaction of *rac*-4-methoxy-phenylalanine after 16 h, with the specific signals of *rac*-4-methoxy-phenylalanine (3.3 min) and of the produced 4-MeO-cinnamic acid (6.2 min).

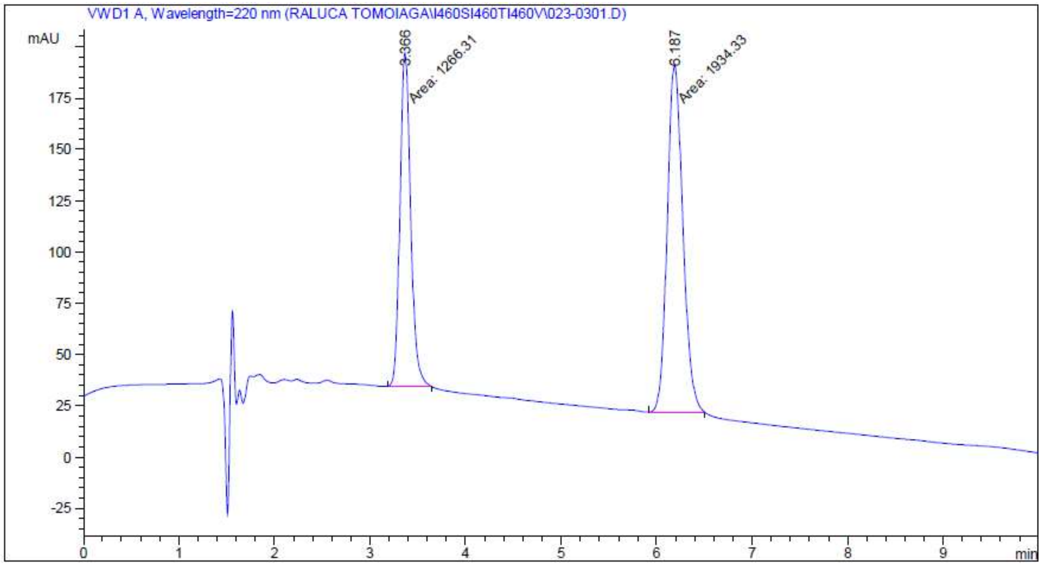

Figure S6. HPLC chromatogram from Gemini NX-C18 column of the isolated I460V-*Pc*PAL catalyzed ammonia elimination reaction of *rac*-4-methoxy-phenylalanine after 16 h, with the specific signals of *rac*-4-methoxy-phenylalanine (3.3 min) and of the produced 4-MeO-cinnamic acid (6.1 min).

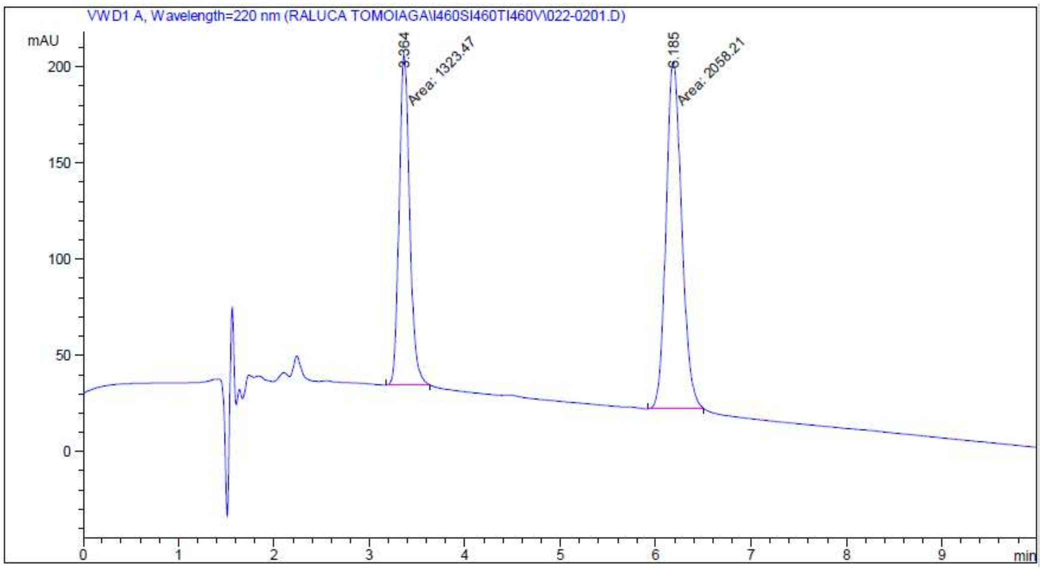

Figure S7. HPLC chromatogram from Gemini NX-C18 column of the isolated I460T-*Pc*PAL catalyzed ammonia elimination reaction of *rac*-4-methoxy-phenylalanine after 16 h, with the specific signals of *rac*-4-methoxy-phenylalanine (3.3 min) and of the produced 4-MeO-cinnamic acid (6.1 min).

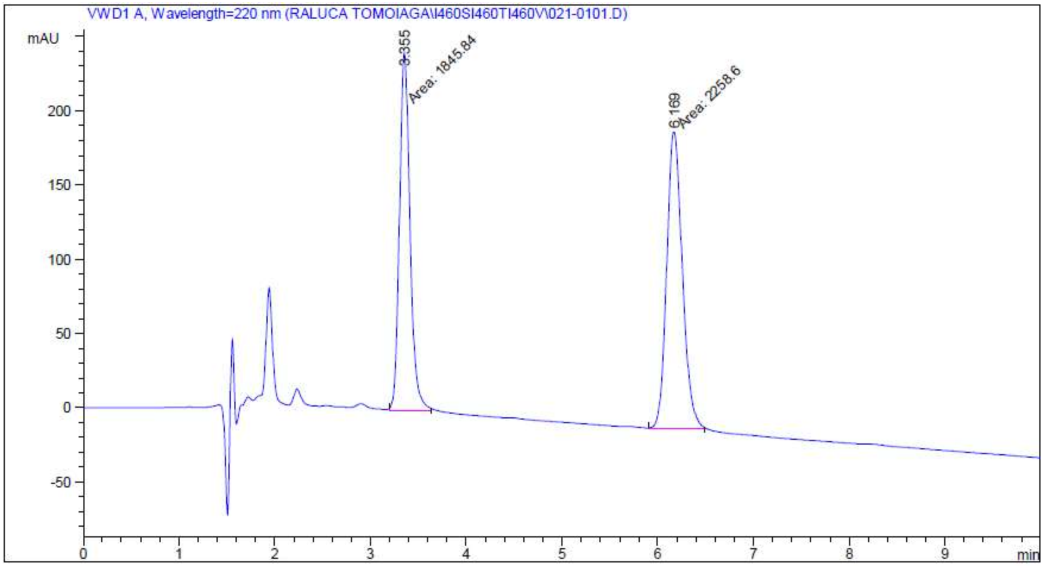

Figure S8. HPLC chromatogram from Gemini NX-C18 column of the isolated I460S-*Pc*PAL catalyzed ammonia elimination reaction of *rac*-4-methoxy-phenylalanine after 16 h, with the specific signals of *rac*-4-methoxy-phenylalanine (3.3 min) and of the produced 4-MeO-cinnamic acid (6.1 min).

5.4. HPLC Method to Determine the Enantiomeric Excess (ee) of p-Methoxy-rac-Phenylalanine

The chiral HPLC separation of the racemic 4-methoxy-phenylalanine was developed using Crownpak CR-I (+) chiral column (150 × 3 mm; 5 μm) and $HClO_4$ (pH = 1.5): acetonitrile as mobile phase, at different flow rate.

Table S7. Retention times of L- and D-4-methoxy-phenylalanine on Crownpak CR-I (+) chiral column.

Compund	Eluent* [% B]	Flow (ml/min)	Retention Time (min)	
			$t_{r\,D}$	$t_{r\,L}$
rac-4-MeO-Phe	**20**	0.4	3.9	7.7

*Mobile phase: A: $HClO_4$ (pH 1.5) / B: ACN, measurements performed at 25° C, Crownpak CR-I (+).

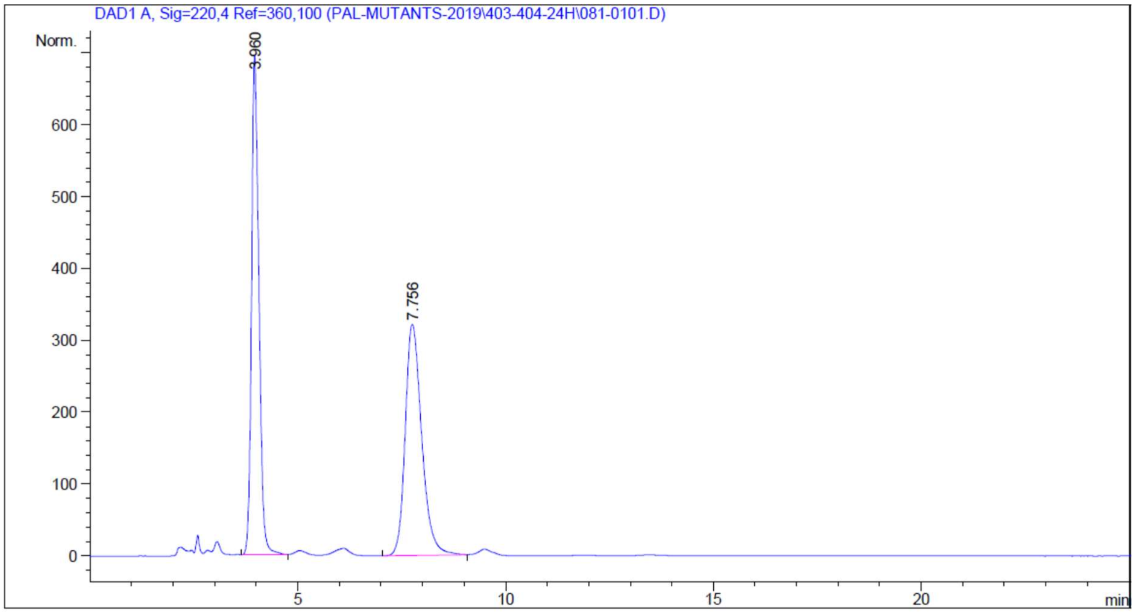

Figure S9. HPLC chromatogram obtained on Crownpak CR-I (+) chiral column for the separation of *rac*-4-methoxy-phenylalanine, with the specific signals of D-4-methoxy-phenylalanine (3.9 min) and L-4-methoxy-phenylalanine (7.7 min).

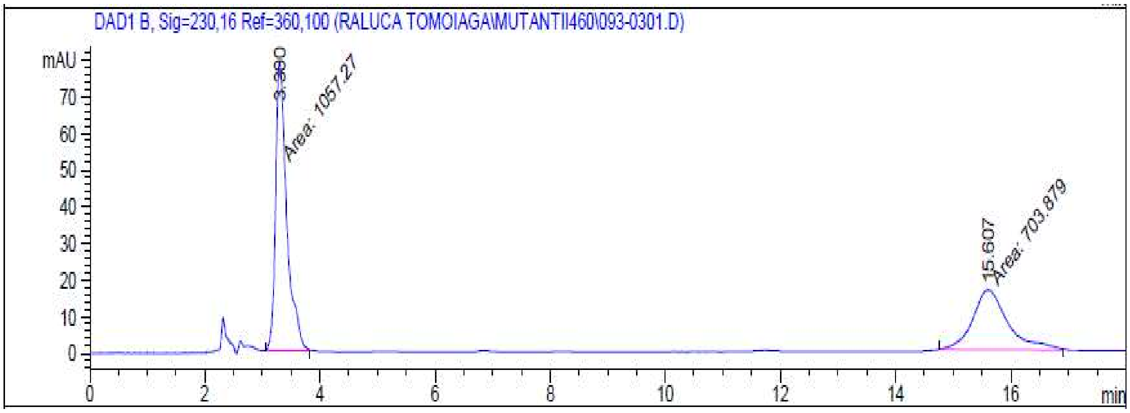

Figure S10. HPLC chromatogram from Crownpak CR-I (+) chiral column for the ammonia elimination of *rac*-4-methoxy-phenylalanine catalyzed by purified I460V-*Pc*PAL, with the specific signals of unreacted D-4-methoxy-phenylalanine (3.39 min) and of the produced 4-MeO-cinnamic acid (15.6 min) – after 4h reaction time.

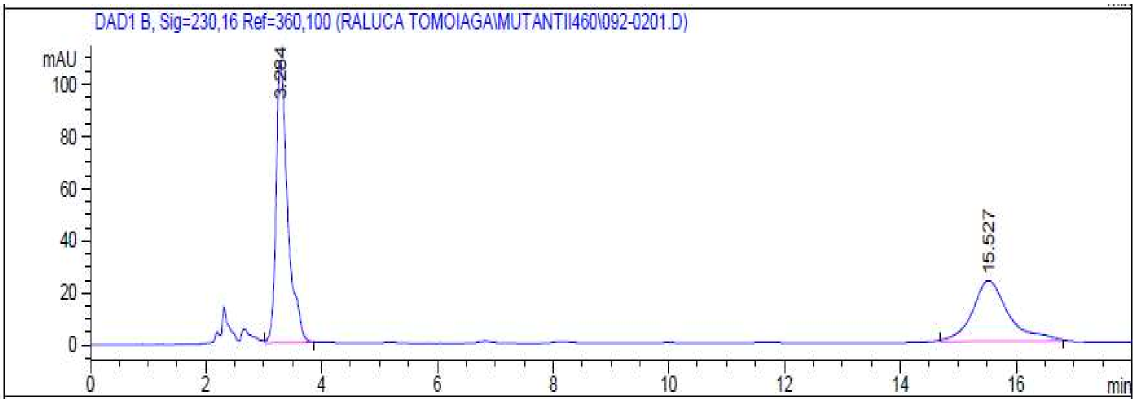

Figure S11. HPLC chromatogram from Crownpak CR-I (+) chiral column for the ammonia elimination of *rac*-4-methoxy-phenylalanine catalyzed by purified I460T-*Pc*PAL, with the specific signals of unreacted D-4-methoxy-phenylalanine (3.3 min) and of the produced 4-MeO-cinnamic acid (15.5 min) – after 4h reaction time.

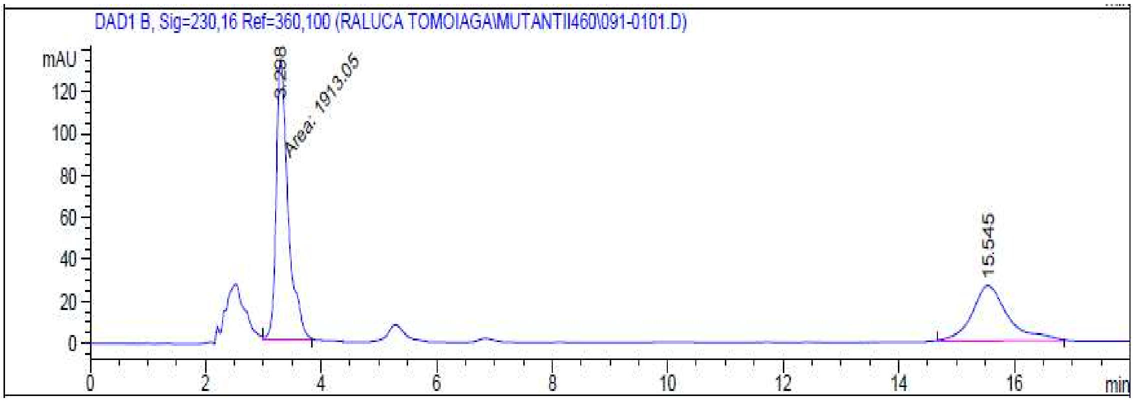

Figure 12. HPLC chromatogram from Crownpak CR-I (+) chiral column for the ammonia elimination of *rac*-4-methoxy-phenylalanine catalyzed by purified I460S-*Pc*PAL, with the specific signals of unreacted D-4-methoxy-phenylalanine (3.3 min) of the produced 4-MeO-cinnamic acid (15.5 min) – after 16 h reaction time.

6. SDS-PAGE Gel from the Purification Steps of I460T-*Pc*PAL and I460S-*Pc*PAL

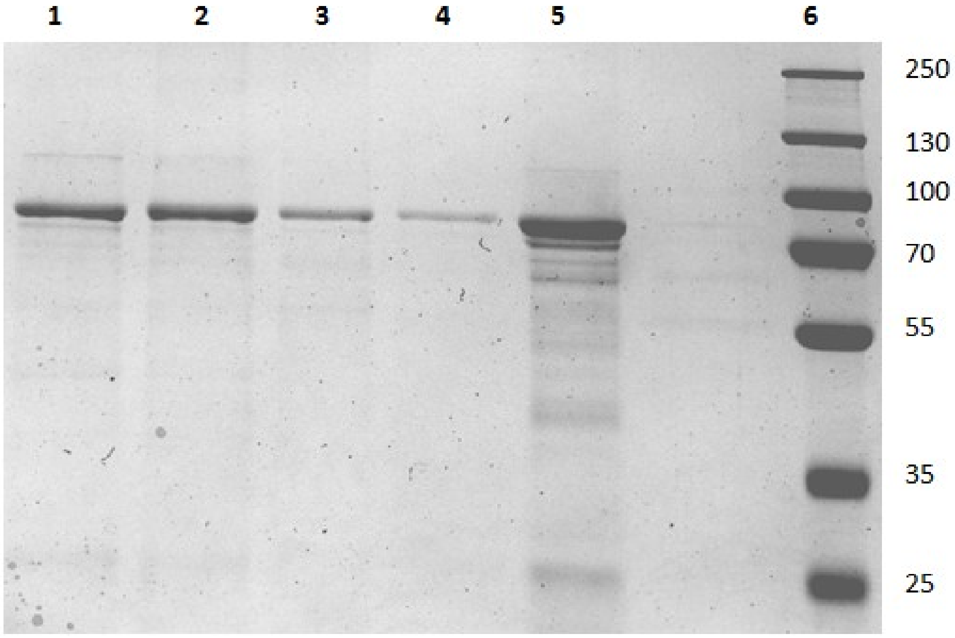

Figure S13. SDS-PAGE gel containing samples from the purification steps of I460T and I460S *Pc*PAL: 1: fraction eluted with 300 mM of imidazole (from Ni-NTA) for I460S; 2: fraction eluted with 300 mM of imidazole (from Ni-NTA) for I460T; 3: fraction eluted with 1M of imidazole (from Ni-NTA) for I460S; 4: fraction eluted with 1 M of imidazole (from Ni-NTA) for I460T; 5: purified I460V (positive control); 6: protein ladder.

7. Enzyme Kinetics

The kinetic measurements were based on UV-spectroscopy by monitoring the production of the cinnamic acid or 4-methoxy cinnamic acid at 290 nm, where the corresponding amino acids (phenylalanine and 4-methoxy-phenylalanine) showed no absorption.

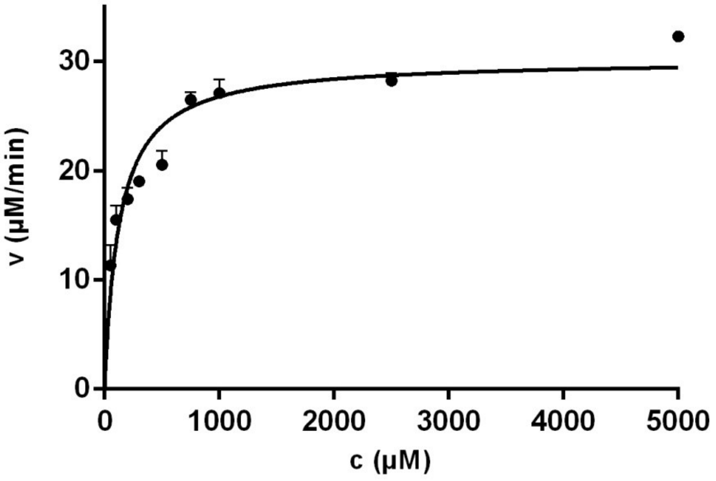

Figure S14. Michaelis-Menten curve for the ammonia elimination from L-Phe catalyzed by I460T-*Pc*PAL; measured in triplicate.

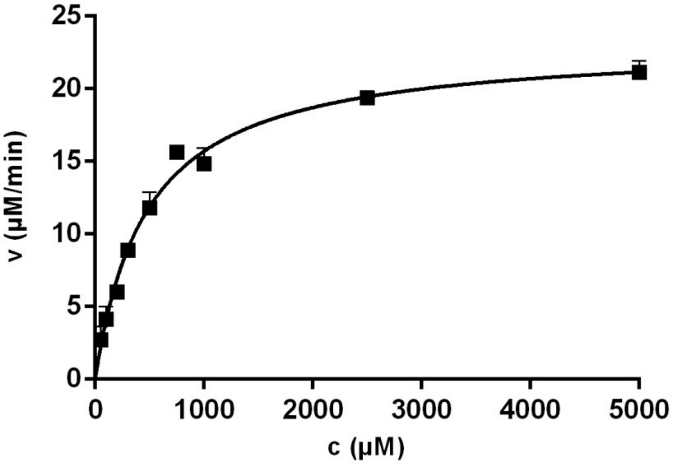

Figure S15. Michaelis-Menten curve for the ammonia elimination from L-Phe catalyzed by I460V-*Pc*PAL; measured in triplicate.

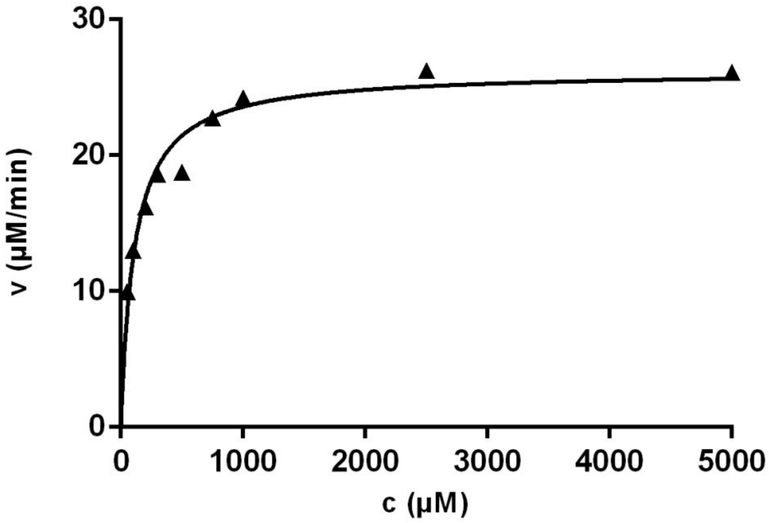

Figure S16. Michaelis-Menten curve for the ammonia elimination from L-Phe catalyzed by *wt-Pc*PAL; measured in triplicate.

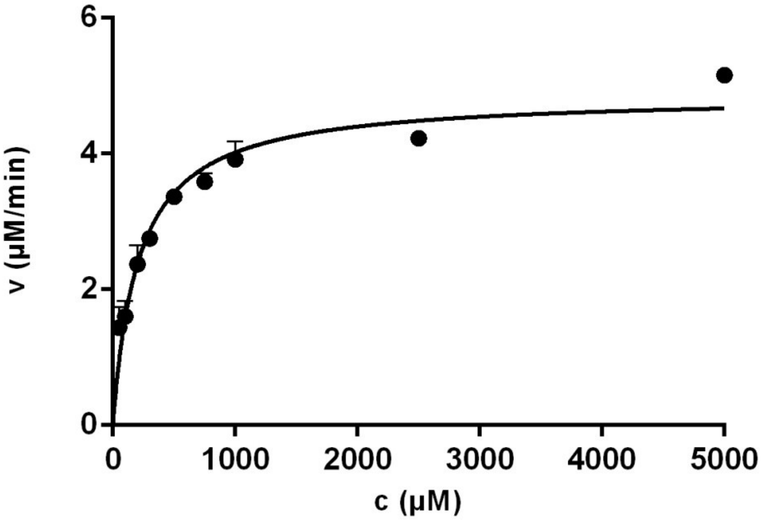

Figure S17. Michaelis-Menten curve for the ammonia elimination from 4-methoxy-*rac*-Phe catalyzed by I460T-*Pc*PAL; measured in triplicate.

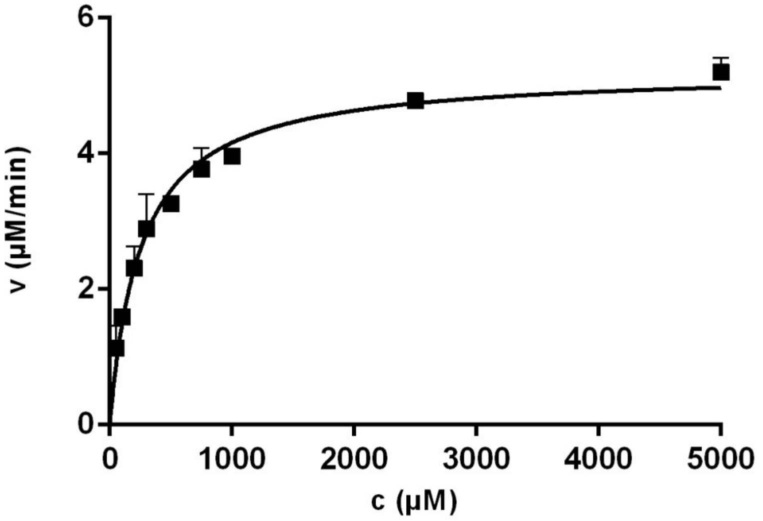

Figure S18. Michaelis-Menten curve for the ammonia elimination from 4-methoxy-*rac*-Phe catalyzed by I460V-*Pc*PAL; measured in triplicate.

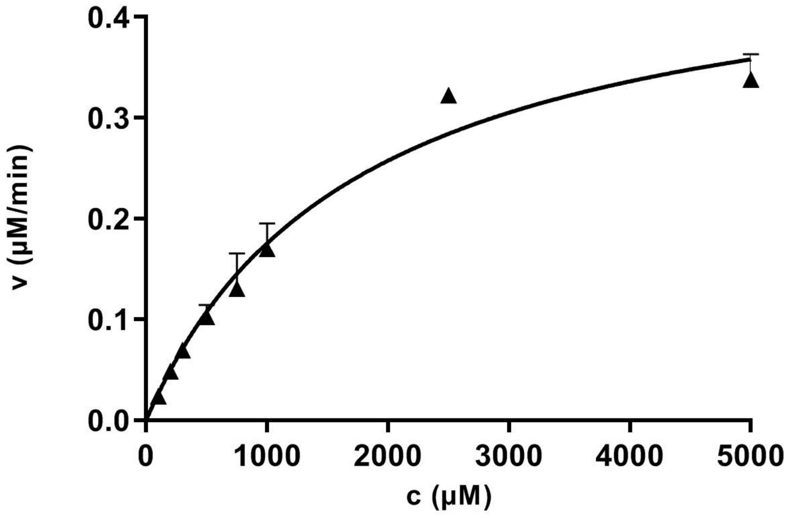

Figure S19. Michaelis-Menten curve for the ammonia elimination from 4-methoxy-*rac*-Phe catalyzed by *wild-type-Pc*PAL; measured in triplicate.

8. Molecular Docking

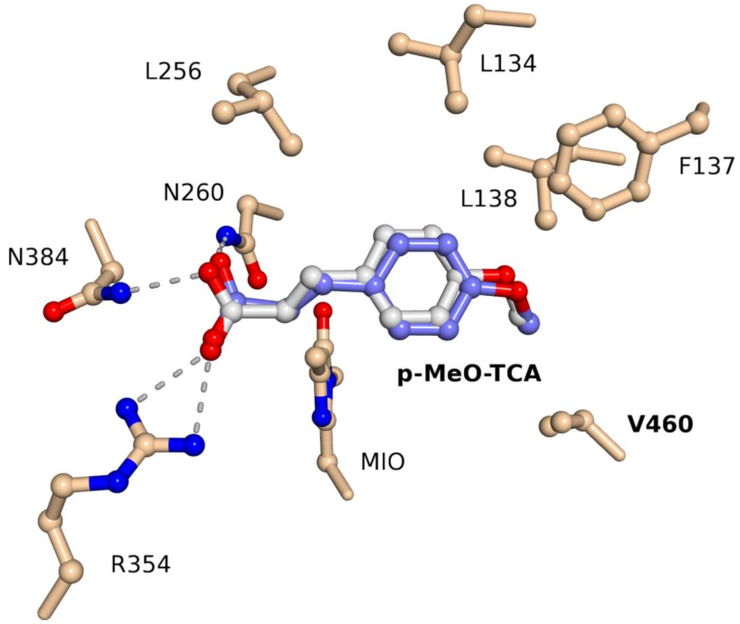

Figure S20. Redocked pose (purple) of the DFT optimized 4-methoxy-TCA in the crystal structure of I460V-*Pc*PAL in comparison with the co-crystallized ligand (gray) retrieved from PDB ID 6RGS.

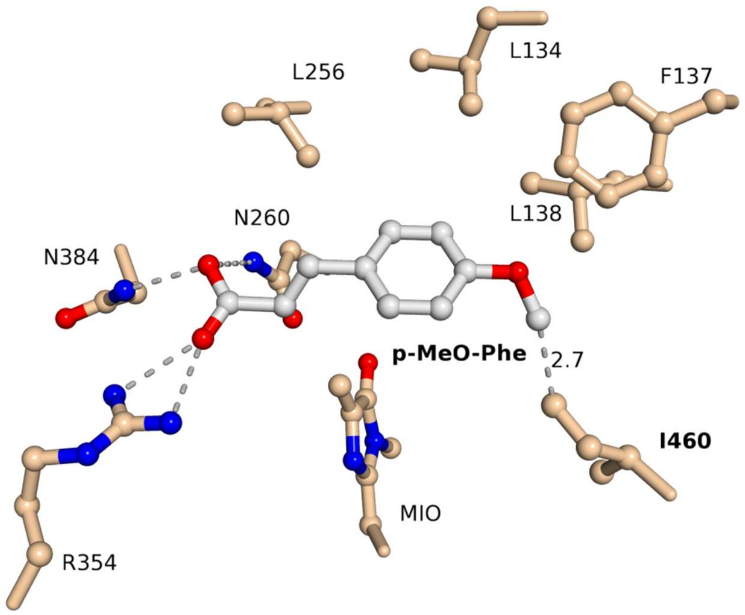

Figure S21. Steric clash exists between the 4-methoxy-TCA (crystal structure) substrate and the I460 residue in the *wt-Pc*PAL.
